# A Prospective Study of Tobacco Smoking and Mortality in Bangladesh

**DOI:** 10.1371/journal.pone.0058516

**Published:** 2013-03-11

**Authors:** Fen Wu, Yu Chen, Faruque Parvez, Stephanie Segers, Maria Argos, Tariqul Islam, Alauddin Ahmed, Muhammad Rakibuz-Zaman, Rabiul Hasan, Golam Sarwar, Habibul Ahsan

**Affiliations:** 1 Department of Population Health, New York University School of Medicine, New York, New York, United States of America; 2 Department of Environmental Health Sciences, Mailman School of Public Health, Columbia University, New York City, New York, United States of America; 3 Department of Health Studies, The University of Chicago, Chicago, Illinois, United States of America; 4 U-Chicago Research Bangladesh, Ltd., Dhaka, Bangladesh; The Ohio State University, United States of America

## Abstract

**Background:**

Limited data are available on smoking-related mortality in low-income countries, where both chronic disease burden and prevalence of smoking are increasing.

**Methods:**

Using data on 20, 033 individuals in the Health Effects of Arsenic Longitudinal Study (HEALS) in Bangladesh, we prospectively evaluated the association between tobacco smoking and all-cause, cancer, and cardiovascular disease mortality during ∼7.6 years of follow-up.

Cox proportional hazards models were used to estimate hazard ratios (HRs) and their 95% confidence intervals (CIs) for deaths from all-cause, cancer, CVD, ischemic heart disease (IHD), and stroke, in relation to status, duration, and intensity of cigarette/bidi and hookah smoking.

**Results:**

Among men, cigarette/bidi smoking was positively associated with all-cause (HR 1.40, 95% CI 1.06 1.86) and cancer mortality (HR 2.91, 1.24 6.80), and there was a dose-response relationship between increasing intensity of cigarette/bidi consumption and increasing mortality. An elevated risk of death from ischemic heart disease (HR 1.87, 1.08 3.24) was associated with current cigarette/bidi smoking. Among women, the corresponding HRs were 1.65 (95% CI 1.16 2.36) for all-cause mortality and 2.69 (95% CI 1.20 6.01) for ischemic heart disease mortality. Similar associations were observed for hookah smoking. There was a trend towards reduced risk for the mortality outcomes with older age at onset of cigarette/bidi smoking and increasing years since quitting cigarette/bibi smoking among men. We estimated that cigarette/bidi smoking accounted for about 25.0% of deaths in men and 7.6% in women.

**Conclusions:**

Tobacco smoking was responsible for substantial proportion of premature deaths in the Bangladeshi population, especially among men. Stringent measures of tobacco control and cessation are needed to reduce tobacco-related deaths in Bangladesh.

## Introduction

Tobacco use is the leading cause of preventable death and disease worldwide and is estimated to kill more than 5 million people each year [1]. According to the World Health Organization (WHO), if current trends continue, by 2030 tobacco use could cause 8 million deaths annually, with more than 80% of these deaths in low- and middle-income countries [2]. While smoking prevalence has declined in many developed countries, it remains high in others and is increasing in low- and middle-income countries [3]. About 80% of the world's smokers now live in low- and middle- income countries. The shift of the tobacco epidemic to the developing world will lead to unprecedented levels of disease and early death in countries where population growth and the potential for increased tobacco use are highest and where health-care services are least available [2].

Cigarette smoking is the most well-known form of tobacco use. Most studies of cigarette smoking and mortality were conducted in Western populations and data are limited in Asian and South Asian. Hookah is another tobacco product with its use rapidly spreading in the United States and Europe [4]. It has been indicated that compared to cigarette smoking, hookah smoke contains 36 times the amount of nicotine and higher concentrations of heavy metals [5], [6]. The investigation of the effects of hookah smoking on mortality is lacking in both Western and Asian populations. The association between cigarette or hookah smoking and mortality may differ from one population to another due to differences in the experience of different stages of epidemiologic transition, demographic profile, life expectancy, as well as distribution of other genetic and environmental risk factors of specific diseases that may interact with effect of smoking [7]. South Asia, where more than half of the world's poor population lives, is also the single largest area on the globe for production and consumption of tobacco products. However, large prospective epidemiologic studies assessing the extent to which smoking characters are related to total and cause-specific mortality, especially cardiovascular disease (CVD) mortality, are lacking. Detailed estimates of smoking-disease association and smoking-attributable mortality may also help target interventions. In the present study, we examined the association of tobacco smoking with all-cause, cancer, and CVD mortality using ∼7.6 years of follow-up data from a large prospective cohort study of individuals chronically exposed to arsenic through drinking water in Araihazar, Bangladesh.

## Materials and Methods

### Study Population

The Health Effects of Arsenic Longitudinal Study (HEALS) is an ongoing prospective, population-based cohort study in Araihazar, Bangladesh [8]. Between October 2000 and May 2002, from a well-defined 25 km^2^ geographical area, we recruited 11,746 men and women (original cohort) who were 1) married (to reduce loss to follow-up) and aged 18–75 years, 2) residents of the study area for at least 5 years prior to recruitment, and 3) primarily drinking water from a local well. During 2006–2008, HEALS was expanded to include an additional 8,287 participants (expansion cohort) following the same methodologies. The overall participation rate was 97%. The participation rate was estimated as the proportion of subjects who agreed to participate among the potential participants we invited to the study [8]. Demographic and lifestyle data for both the original and expansion cohort participants were collected using a standardized questionnaire. Trained study physicians measured height and weight using a locally manufactured tape measure and a Misaki (Okaka, Japan) scale (calibrated weekly), respectively. Both height and weight were measured three times at baseline and averaged [9]. Trained clinicians measured blood pressure with an automatic sphygmomanometer [10], [11]. Although the original aim of the study was to investigate health effects of arsenic exposure, population-based studies can be used to provide epidemiologic data on health effects of smoking that may be more generalizable.

Follow-up in-person interviews at two-year intervals were conducted by trained physicians following the same procedures used in the baseline interview. Between the biennial follow-up visits, passive follow-up is conducted, as participants with health conditions would visit the field clinic which was established exclusively for the cohort participants to receive medical diagnoses and treatments [8], and the relevant data are collected. Informed consent was obtained from study participants and the study procedures were approved by the Ethical Committee of the Bangladesh Medical Research Council and the Institutional Review Boards of Columbia University and the University of Chicago. The present study included follow up data from baseline to October 9, 2011.

### Assessment of Mortality

Details of the methods for the assessment of causes of death are described elsewhere [9], [12], [13]. Briefly, we adapted a validated verbal autopsy procedure, developed by the International Centre for Diarrhea Disease Research, Bangladesh, in collaboration with the WHO, to ascertain the causes of death. During the follow-up, upon receipt of a death reported by family or neighbors, a study physician and a trained social worker administered the verbal autopsy form to the next of kin. Medical records and death certificates were collected and reviewed monthly by an outcome assessment committee, consisting of physicians and consulting medical specialists. Causes of deaths were coded according to the WHO classification [14] and the International Classification of Diseases, 10th Revision (ICD-10) [15]. The International Centre for Diarrhea Disease Research, Bangladesh has used this method to ascertain causes of deaths since 1971 [16], [17] and documented an overall 95% specificity, with a 85% sensitivity for cancer deaths and 85% sensitivity for deaths due to CVD [18].

### Assessment of Tobacco Smoking Variables

At baseline, detailed information on smoking of tobacco products was collected. Details of smoking cigarettes and bidis (filterless locally produced cigarettes) were asked together, including information on past or current use, duration of use, age at start, number of sticks per day, and age at quitting. A separate set of questions were asked for hookah smoking (tobacco smoking using waterpipes). We observed a high correlation between hookah and cigarette/bidi smoking, such that 98% of the 2,005 ever smokers of hookah were ever smokers of cigarette/bidi. Although cigarettes and bidis frequently are sold individually in Bangladesh, we calculated “pack-years” (product of sticks of cigarette/bidi per day and years of smoking, divides by 20) for ease in comparison with other studies. Similarly, a “time-years” index (product of times per day and years of smoking) was calculated for hookah smoking.

### Statistical Analyses

Person years of follow-up were calculated from baseline to the date of death from any cause (for those who died) or to October 9, 2011, the date of last death observed in the follow-up at the time of data analyses (for those who were alive). Cox proportional hazards models were used to estimate hazard ratios (HRs) and their 95% confidence intervals (CIs) for deaths from all-cause, cancer, CVD, ischemic heart disease (IHD), and stroke, in relation to status, duration, and intensity of cigarette/bidi and hookah smoking. Survival curves for overall survival were graphed by cigarette/bidi smoking status among men and women. We evaluated the effect of age at starting cigarette/bidi smoking and time since quitting smoking on mortality from all-cause, cancer, and IHD among men only, as the data were limited in women. All the analyses were stratified by sex, as the prevalence of smoking is different in men and women. HRs were adjusted for baseline age (years), body mass index (BMI; kg/m^2^), and educational attainment (years). Analyses were done with additional adjustments of other potential confounders such as betel quid chewing and arsenic exposure or potential intermediate factors such as systolic blood pressure and diabetes. Results were similar and therefore are not shown. Additional analyses were conducted to control for cigarette/bidi smoking in the analyses of hookah smoking, and *vice versa*. However, almost all the hookah smokers (99.1%) were also cigarette/bidi smokers in our study population, and therefore it was not possible to estimate HRs associated with hookah smoking among nonsmokers of cigarette/bidi or to assess additive interaction between hookah and cigarette/bidi smoking. Additional stratification by age, education, BMI, and betel quid chewing status did not suggest any subgroup-specific associations (all *P* for interaction were >0.10) and therefore results are not shown. We calculated the population attributable fraction (PAF) for all-cause mortality associated with ever cigarette/bidi or hookah smoking and smoking intensity using adjusted HRs estimated from Cox proportional hazards models. PAF was calculated as follows [19]:
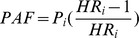



Where *P_i_* is the proportion of all-cause deaths in the *i*th smoking category and *HR_i_* is the adjusted hazard ratio associated with the *i*th smoking category (relative to nonsmokers). All analyses were done with the SPSS 19.0 software (SPSS Inc., Chicago, IL).

## Results

We observed 151, 641 person years during an average of 7.6 years of follow-up. There were 734 deaths, of which 128 were from cancers and 308 were from CVD, accounting for 60% of mortality. Among the 308 deaths from CVD, 119 were from IHD and 129 were from stroke. The underlying causes of death and ICD-10 codes were shown in Table S1.

**Figure 1:**
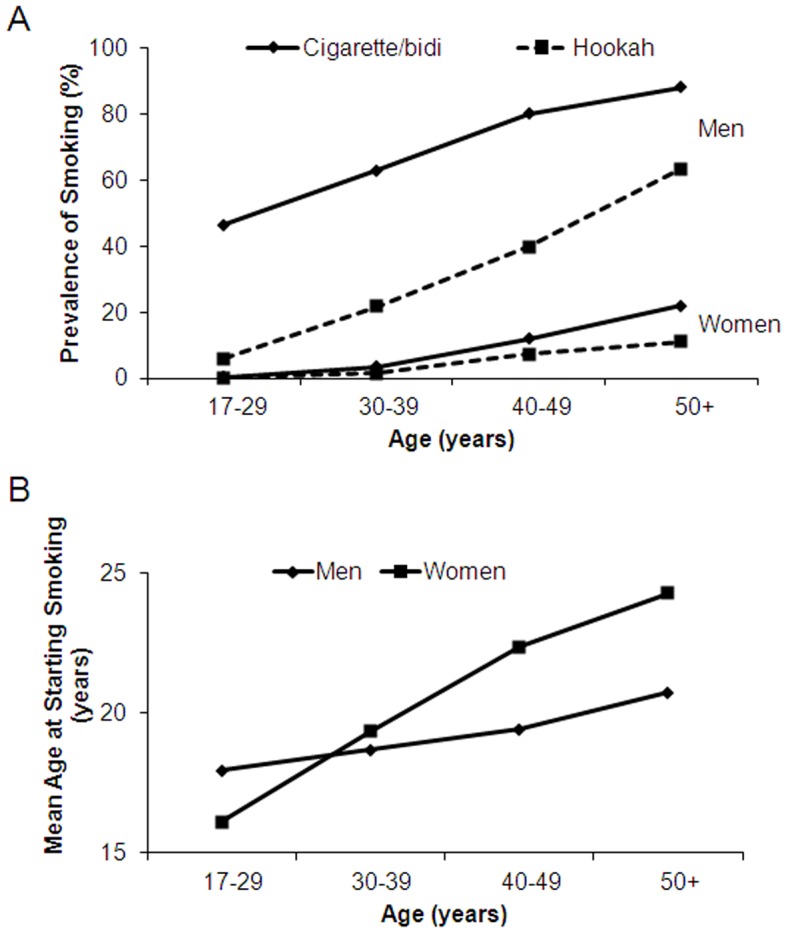
Prevalence of smoking and age at starting smoking. (**A**) Prevalence of cigarette/bidi smoking and hookah smoking by age groups (17–29, 30–39, 40–49, and 50+) among men and women. (**B**) Mean age at starting cigarette/bidi smoking by age groups among men and women.

The prevalence of cigarette/bidi smoking among men rose with age, from 47% among those between 17 and 29 years to 88% among those over 50 years. The age-specific prevalence of smoking among women rose from 0.4 to 22%. The pattern of hookah smoking is similar as that of cigarette/bidi smoking, although the age-specific prevalence is much lower among men (Figure 1A). Interestingly, in both men and women, younger smokers of cigarette/bidi started smoking at an earlier age, with smokers aged 17–29 years starting smoking before age 18 (Figure 1B). The prevalence of cigarette/bidi smoking according to other baseline characteristics is shown in Table 1. Among both men and women, cigarette/bidi smoking was more common among those without primary education or with a lower BMI than among other participants. The prevalence of cigarette/bidi smoking was higher among ever hookah smokers and ever chewers of betel quid, compared with never hookah smokers and never chewers among both men and women. Women who had a higher systolic blood pressure were more likely to be ever cigarette/bidi smokers whereas men who had a lower diastolic blood pressure were more likely to be ever cigarette/bidi smokers.

**Table 1:** Prevalence of ever cigarette/bidi smoking by baseline characteristics.^a^

	Men		Women	
	No. (%)	*P* Value^b^	No. (%)	*P* Value^b^
Education (years)				
No	2622 (80.5)	<0.001	585 (10.7)	<0.001
1–5	1790 (69.0)		94 (2.7)	
6–9	722 (63.5)		5 (0.3)	
≥10	689 (59.1)		2 (0.2)	
Religion				
Muslim	5532 (71.2)	0.04	675 (6.0)	<0.001
Hindu/Other	297 (76.0)		11 (2.0)	
Body mass index (kg/m^2^)				
<18.5	2883 (80.1)		421 (10.0)	<0.001
18.5–24.9	2633 (65.7)		240 (3.7)	
>24.9	218 (50.2)		18 (1.9)	
Hookah smoking				
Never	1949 (60.6)	<0.001	247 (3.8)	<0.001
Ever	1803 (99.1)		168 (90.8)	
Betel quid chewing				
Never	3076 (61.4)	<0.001	126 (1.6)	<0.001
Past	255 (88.5)		31 (17.5)	
Current	2486 (87.2)		529 (14.4)	
Systolic blood pressure				
<140	5263 (71.5)	0.29	589 (5.5)	<0.001
≥140	471 (69.6)		91 (9.0)	
Diastolic blood pressure				
<90	5345 (72.2)	<0.001	609 (5.8)	0.97
≥90	388 (61.1)		70 (5.8)	
History of diabetes				
Yes	110 (66.3)	0.16	7 (4.0)	0.30
No	5567 (71.3)		668 (5.8)	

a Data were missing on cigarette/bidis smoking for 8 subjects; on education for 11 subjects; on religion for 1 subject; on body mass index for 281 subjects; on hookah smoking for 8295 subjects; on betel quid chewing for 34 subjects; on systolic blood pressure for 260 subjects; on diastolic blood pressure for 268 subjects; and on history of diabetes for 321 subjects.

b *P*-value from the chi-square test or *t*-test.

**Figure 2:**
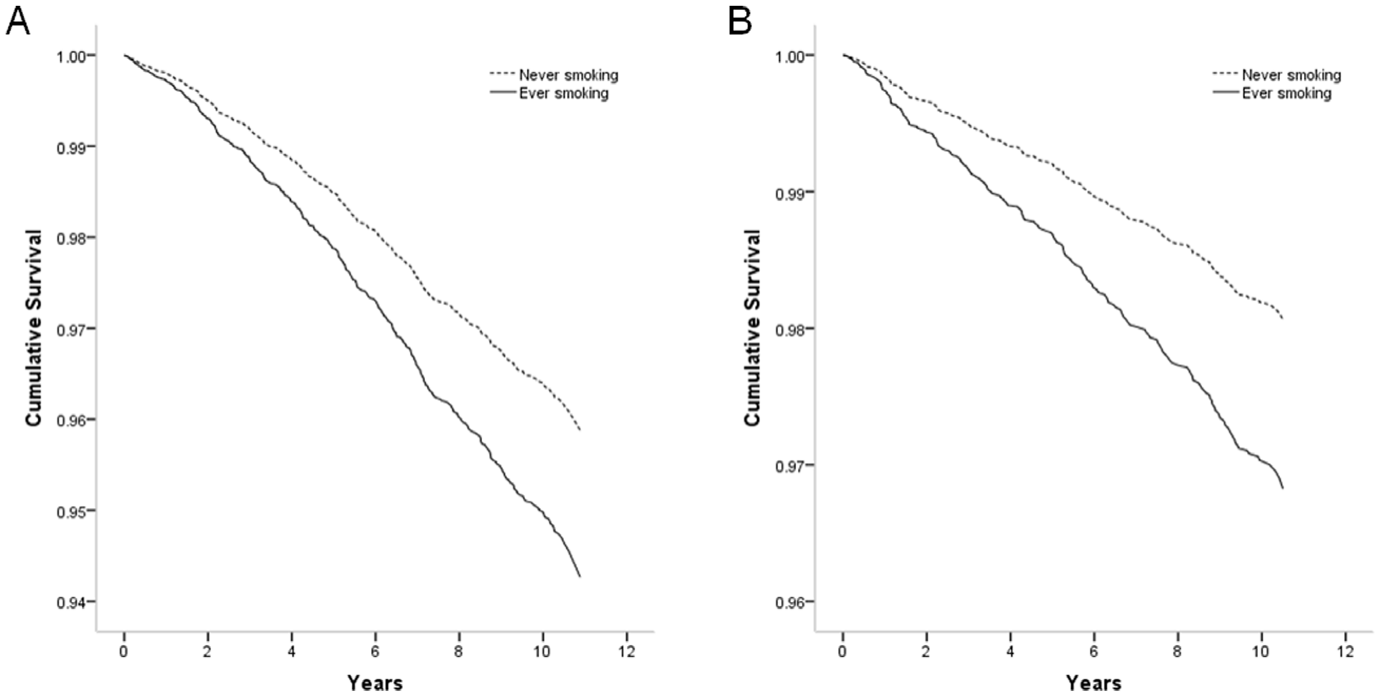
Survival curves for overall survival by never and ever cigarette/bidi smoking. (**A**) Overall survival among men. (**B**) Overall survival among women. Cox proportional hazards regression analyses were adjusted for baseline age (years), body mass index, and educational attainment (years).

In men, there was an increased risk of all-cause and cancer mortality in ever, past, and current smokers of cigarette/bidi, compared with never smokers (Table 2). For both all-cause and cancer mortality, there was a highly significant trend of increasing mortality with increasing intensity of tobacco consumption. Ever smokers who had smoked cigarette/bidi more than 10 sticks per day, had smoked for more than 20 years, or had accumulated more than 10 pack-years were 1.4–1.8 times more likely to die from all-cause and 3.3–4.2 times more likely to die from cancer. Similar but much weaker associations were observed of hookah smoking with all-cause and cancer mortality. On the other hand, there was no apparent association between either cigarette/bidi or hookah smoking and mortality from overall CVD. In women, there was an increased risk of all-cause mortality among ever (HR 1.65, 1.16 2.36) and past cigarette/bidi smokers (HR 1.92, 1.21 3.05), as well as among ever hookah smokers (HR 2.81, 1.78 4.43). The number of deaths from cancer in women, however, was not enough for us to evaluate the association between tobacco smoking and cancer mortality. The effect estimates for cigarette/bidi and hookah smoking were in general similar although somewhat weaker after mutual adjustment. For instance, the HR for the risk of death from cancer in relation to ever cigarette/bidi smoking changed from 1.70 (95% CI 1.00 2.91) to 1.58 (95% CI 0.84 2.96) in overall and from 2.91 (95% CI 1.24 6.80) to 2.54 (95% CI 1.06 6.12) in men, after adjustment for hookah smoking. The HR for cancer deaths associated with ever hookah smoking changed from 1.38 (95% CI 0.86 2.22) to 1.21 (95% CI 0.74 1.99) in overall and from 1.30 (95% CI 0.78 2.18) to 1.07 (95% CI 0.63 1.80) in men, after adjustment for cigarette/bidi smoking. Figure 2 shows the survival curves for overall survival by cigarette/bidi smoking status among men and women. The probability of survival was significantly lower for ever cigarette/bidi smokers than for never smokers in both men and women.

**Table 2:** Association between smoking and mortality from all-cause, cancer, and cardiovascular diseases.

	Person years	All-cause		Cancer			CVD	
		No. of deaths	Hazard ratio (95% confidence interval)^a^	No. of deaths	Hazard ratio (95% confidence interval)^a^		No. of deaths	Hazard ratio (95% confidence interval)^a^
Men								
Cigarette/bidis smoking								
Nonsmokers^b^	17294	62	1.00	6		1.00	35	1.00
Ever smokers	45125	439	1.40 (1.06–1.86)	82		2.91 (1.24–6.80)	193	1.18 (0.81–1.71)
Past smokers	7157	113	1.48 (1.07–2.05)	19		2.94 (1.14–7.57)	43	0.89 (0.56–1.42)
Current smokers	37969	326	1.38 (1.04–1.84)	63		2.90 (1.23–6.84)	150	1.30 (0.88–1.91)
Number of cigarette smoked per day^c^								
1–10	26017	231	1.31 (0.98–1.76)	38		2.43 (1.01–5.88)	108	1.19 (0.80–1.78)
11–20	12770	128	1.47 (1.07–2.01)	26		3.28 (1.33–8.11)	53	1.13 (0.73–1.76)
21–125	6290	78	1.55 (1.09–2.19)	18		4.01 (1.55–10.4)	32	1.21 (0.74–1.98)
*P* for trend			0.01			0.002		0.61
Years of smoking^c^								
1–20	17961	95	1.28 (0.92–1.78)	19		2.64 (1.04–6.68)	36	0.94 (0.58–1.52)
21–30	11907	116	1.44 (1.05–1.98)	25		3.40 (1.37–8.42)	54	1.24 (0.80–1.92)
31–60	7753	194	1.80 (1.31–2.48)	34		3.73 (1.46–9.53)	88	1.40 (0.90–2.15)
*P* for trend			<0.001			0.007		0.06
Pack–years^c^								
1–10	17050	109	1.21 (0.88–1.67)	15		1.82 (0.70–4.76)	54	1.17 (0.75–1.81)
11–30	10495	136	1.70 (1.24–2.33)	29		4.20 (1.70–10.4)	52	1.18 (0.75–1.84)
31–238	10029	158	1.57 (1.15–2.14)	34		4.07 (1.65–10.0)	72	1.25 (0.81–1.91)
*P* for trend			0.001			<0.001		0.35
Hookah smoking								
Nonsmokers^d^	31988	192	1.00	34		1.00	88	1.00
Ever smokers	17280	258	1.15 (0.93–1.43)	47		1.30 (0.78–2.18)	115	1.20 (0.87–1.67)
Past smokers	15871	231	1.12 (0.90–1.41)	40		1.19 (0.70–2.02)	106	1.22 (0.88–1.71)
Current smokers	1409	27	1.46 (0.94–2.25)	7		2.51 (1.08–5.82)	9	1.00 (0.46–2.18)
Times smoked per day^c^								
≤5	10419	122	1.03 (0.80–1.32)	20		1.04 (0.57–1.89)	57	1.11 (0.77–1.60)
>5	6829	136	1.35 (1.05–1.76)	27		1.75 (0.97–3.18)	58	1.36 (0.92–2.01)
*P* for trend			0.03			0.07		0.13
Years of smoking^c^								
≤5	8786	91	1.04 (0.80–1.36)	18		1.23 (0.67–2.25)	41	1.13 (0.76–1.68)
>5	8397	167	1.27 (0.99–1.63)	29		1.39 (0.78–2.50)	74	1.29 (0.89–1.87)
*P* for trend			0.06			0.27		0.18
Time–years^c^								
≤30	9285	101	1.07 (0.82–1.38)	17	1.09 (0.59–2.02)		50	1.25 (0.86–1.82)
30	7897	157	1.26 (0.98–1.63)	30	1.58 (0.88–2.85)		65	1.17 (0.80–1.72)
P for trend			0.07			0.13		0.40
Women								
Cigarette/bidis smoking								
Nonsmokers^b^	83950	187	1.00	—		—	63	1.00
Ever smokers	5203	45	1.65 (1.16–2.36)	—		—	16	1.45 (0.80–2.60)
Past smokers	2179	23	1.92 (1.21–3.05)	—		—	7	—
Current smokers	3024	22	1.45 (0.91–2.30)	—		—	9	—
Hookah smoking								
Nonsmokers^d^	65852	150	1.00	—		—	52	1.00
Ever smokers	1733	24	2.81 (1.78–4.43)	—		—	8	2.08 (0.96–4.49)

a Adjusted for baseline age (years), body mass index, and educational attainment.

b Non-cigarette/bidi smokers as the reference group.

c Categories in ever smokers were based on meaningful cut points for easy interpretation.

d Non-hookah smokers as the reference group.

Further analyses on subtypes of CVD showed that there was an increased risk of death from IHD among both ever (HR 1.68, 0.94 2.99) and current cigarette/bidi smokers (HR 1.94, 1.08 3.49) in men, with a stronger association for current smokers (Table 3). In addition, ever smokers who had smoked for more than 30 years, or had accumulated more than 30 pack-years were 2.0–2.1 times more likely to die from IHD. Ever hookah smokers with higher daily intensity (>5 times/day) were 1.96 (95% CI 1.05 3.63) times more likely to die from IHD than never smokers. In women, ever cigarette/bidi smoking was related to a non-significant increase in risk of IHD was (HR 2.55, 0.86 7.57).

**Table 3:** Association between smoking and mortality from IHD and stroke.

	Person years	IHD		Stroke	
		No. of deaths	Hazard ratio (95% confidence interval)^a^	No. of deaths	Hazard ratio (95% confidence interval)^a^
Men					
**Cigarette/bidi smoking**					
Nonsmokers^b^	17294	15	1.00	14	1.00
Ever smokers	45125	86	1.68 (0.94–2.99)	79	0.91 (0.50–1.64)
Past smokers	7157	17	1.03 (0.49–2.18)	18	0.70 (0.34–1.44)
Current smokers	37969	69	1.94 (1.08–3.49)	61	1.01 (0.55–1.86)
Number of cigarette smoked per day^c^					
1–10	26017	47	1.65 (0.90–3.05)	44	0.92 (0.49–1.72)
11–20	12770	26	1.75 (0.90–3.40)	19	0.79 (0.39–1.60)
21–125	6290	13	1.64 (0.76–3.55)	16	1.10 (0.53–2.29)
* P* for trend			0.20		0.93
Years of smoking^c^					
1–20	17961	16	1.18 (0.57–2.46)	14	0.83 (0.39–1.76)
21–30	11907	26	1.71 (0.87–3.35)	20	1.04 (0.52–2.07)
31–60	7753	33	2.13 (1.06–4.29)	44	1.06 (0.56–2.03)
* P* for trend			0.02		0.62
Pack–years^c^					
1–10	17050	21	1.37 (0.69–2.73)	26	1.16 (0.60–2.26)
11–30	10495	21	1.51 (0.74–3.06)	23	0.98 (0.49–1.94)
31–238	10029	33	1.99 (1.03–3.85)	29	0.89 (0.46–1.72)
* P* for trend			0.04		0.48
**Hookah smoking**					
Nonsmokers^d^	31988	41	1.00	38	1.00
Ever smokers	17280	43	1.45 (0.86–2.43)	53	0.91 (0.56–1.46)
Times smoked per day^c^					
≤5	11920	19	1.18 (0.64–2.16)	27	0.90 (0.53–1.53)
>5	7050	24	1.96 (1.05–3.63)	26	0.92 (0.52–1.64)
* P* for trend			0.04		0.77
Years of smoking^c^					
≤5	9735	21	1.74 (0.98–3.10)	14	0.70 (0.37–1.32)
>5	9181	22	1.19 (0.63–2.25)	39	1.07 (0.63–1.81)
* P* for trend			0.47		0.76
Time–years^c^					
≤30	10494	21	1.63 (0.92–2.88)	20	0.90 (0.51–1.58)
>30	8412	22	1.28 (0.67–2.44)	33	0.92 (0.53–1.60)
* P* for trend			0.36		0.77
Women					
**Cigarette/bidis smoking**					
Nonsmokers^b^	17294	11	1.00	30	1.00
Ever smokers	45125	6	2.55 (0.86–7.57)	6	0.98 (0.39–2.46)

a Adjusted for baseline age (years), body mass index, and educational attainment.

b Non-cigarette/bidi smokers as the reference group.

c Categories in ever smokers were based on meaningful cut points for easy interpretation.

d Non-hookah smokers as the reference group.

The proportion of all-cause deaths attributable to ever cigarette/bidi smoking was 25.0% in men and 7.6% in women (Table 4). The PAF was 4.1%, 12.0%, and 12.3% for light, moderate, and heavy cigarette/bidi smokers. Ever hookah smoking was a cause of 7.5% and 8.9% of all-cause deaths in men and women, respectively.

**Table 4:** Population attributable fraction for all-cause mortality by smoking.

	Proportion of deaths (%)	Population attributable fraction	
		%	95% confidence interval
Men			
Ever cigarette/bidi smoking	87.6	25.0	3.8–40.5
Smoking intensity (pack-years)			
1–10	23.4	4.1	−2.3–9.4
11–30	29.2	12.0	4.1–16.7
31–238	34.0	12.3	3.2–18.1
Ever hookah smoking	57.3	7.5	−3.5–17.2
Women			
Ever cigarette/bidi smoking	19.4	7.6	1.9–11.2
Ever hookah smoking	13.8	8.9	3.8–10.7

In men, the HR for all-cause mortality among current cigarette/bidi smokers who started smoking at age 18 years or younger were 1.64 (1.21 2.22), 43% higher than that among those starting at or after 18 years (HR 1.15, 0.84 1.58, Table 5). Similarly, earlier starting age of smoking was associated with a greater risk of mortality due to cancer and IHD, and the association was stronger compared to that for all-cause mortality. For mortality from all-cause and cancer, while the risk was reduced 5 years after quitting smoking compared with continuing to smoke, there was a non-significant increase in risk within 5 years of quitting smoking. For IHD mortality, there was a significant downward trend with increasing years since quitting, with a significant 58% reduction in the risk after 5 or more years since quitting smoking compared with continued smoking (HR 0.42, 0.19 0.94).

**Table 5:** Association of age at starting cigarette/bidi smoking and time since quitting smoking with mortality from all-cause, cancer, and IHD among men.

	Person years	All-cause		Cancer		IHD	
		No. of deaths	Hazard ratio (95% confidence interval)^a^	No. of deaths	Hazard ratio (95% confidence interval)^a^	No. of deaths	Hazard ratio (95% confidence interval)^a^
Never smokers^b^	17295	62	1.00	6	1.00	15	1.00
Starting age, years^c^							
≤18	19936	182	1.64 (1.21–2.22)	35	3.46 (1.42–8.43)	37	2.21 (1.17–4.17)
>18	18006	142	1.15 (0.84–1.58)	28	2.54 (1.02–6.31)	32	1.77 (0.92–3.41)
*P* for trend			<0.001		0.005		0.02
Current smokers^b^	37969	326	1.00	63	1.00	69	1.00
Years since quitting^d^							
≤5	3421	52	1.17 (0.87–1.58)	10	1.24 (0.63–2.43)	8	0.73 (0.35–1.54)
>5	3495	58	0.97 (0.72–1.31)	9	0.88 (0.43–1.82)	9	0.42 (0.19–0.94)
Nonsmokers	17295	62	0.72 (0.54–0.96)	6	0.34 (0.15–0.81)	15	0.51 (0.28–0.92)
*P* for trend			0.06		0.03		0.006

a Adjusted for baseline age (years), BMI, and educational attainment.

b Non-cigarette/bidi smokers as the reference group.

c Among current smokers.

d Among past smokers.

## Discussion

In the present study, we found that cigarette/bidi or hookah smoking was significantly associated with an increased risk of death from all-cause, cancer, and IHD. There was a highly significant trend of increasing all-cause and cancer mortality with increasing cigarette/bidi consumption. The deaths attributable to cigarette/bidi smoking were much greater in men than in women, with cigarette/bidi smoking accounting for 25.0% of overall mortality in men and 7.6% in women. Significantly increased risk associated with earlier age at initiation of cigarette/bidi smoking was observed for all-cause, cancer, and IHD mortality. The excess risk reduced to the level of never smokers after 5 years or more of quitting smoking for IHD mortality but not for cancer mortality. To the best of our knowledge, the present study is the first prospective study that reports a positive association between cigarette/bidi smoking and IHD mortality in South Asians, and the first that reports a benefit of quitting smoking on IHD mortality.

The positive association between tobacco smoking and all-cause mortality among both men and women is clearly demonstrated in Western and Asian populations [20]–[26]. Previous studies in India have also provided evidence of excess all-cause mortality associated with smoking, with relative risks ranging from 1.3 to 2.1 [27]–[31] and a significant dose–response relationship for frequency and duration [27]–[29], [31] observed in men. Smoking in women is relatively uncommon in India, however, similar relative risk ranging from 1.3 to 2.0 was reported [27], [28], [31]. We also found a higher risk among smokers in comparison with never users for all-cause mortality, with the HR comparable to those previously reported in Indians. Our lower risks associated with smoking than those reported from Western populations might be due to the lower intensity of cigarette/bidi smoking in the past (mean 12 sticks/day), the older age of smoking initiation (mean 20 years) compared with Western smokers [32], or different distribution of risk factors that may interact with the effect of smoking. The risk of all-cause mortality was also higher for hookah users compared to non-users. It has been reported that water pipe smoking delivers the addictive drug nicotine and is at least as toxic as cigarette smoke [33], and hookah smokers are at risk for the same categories of diseases associated with cigarette smoking [34]. Although the prevalence of hookah smoking has reduced over the years, our data suggested that the use was more common in women and hookah smoking accounted for more deaths than cigarette/bidi smoking did in women.

Cohort studies consistently support an increased risk of cancer mortality associated with tobacco smoking [25], [26], [29]. Our overall HR of 1.7 for ever smokers was similar to estimates from studies conducted in India, which reported HRs of 1.6–2.0 [29], [35], [36]. We found that current hookah smokers were 2.5 times more likely to die from cancer among men. The literature on the effects of hookah smoking on cancer is limited. Given that most of hookah smokers (99.1%) are also cigarette/bidi smokers in our study population, it is difficult to isolate the effects of hookah smoking from that of cigarette/bidi smoking. We also have a limited sample size to evaluate cancer specific mortality. Further larger studies on the associations between hookah smoking and risk of cause-specific cancer mortality are warranted.

Current cigarette/bidi smoking was significantly associated with an increased risk of IHD mortality among men, a finding consistent with previous reports from Japan [37], Korea [38], and Singapore [25]. A cohort study from India reported a RR of 1.17 (0.99 1.39) for IHD associated with smoking in men [28], which was much lower than the HR of 1.68 from our study (0.94 2.99). Retrospective and case-control studies from India, however, reported significant associations between ever smoking and mortality from heart disease and other vascular disease with RRs ranging from 1.6–1.8 [30], [31]. On the other hand, there was no apparent association between tobacco smoking and stroke mortality, consistent with many, [25], [37], [39] but not all, [30], [31], [40], [41] previous studies from Asia including India. The effects of smoking on stroke have been inconsistent by stroke subtypes. For instance, the association between smoking and subarachnoid hemorrhage is stronger compared with other types of stroke [40], [42], [43] while most of the previous studies did not show excess mortality from subtypes of ischemic stroke such as embolic brain infarction among smokers [44], [45]. Further studies are warranted to elucidate the association between smoking and stroke subtypes in South Asians.

If the associations we observed in our study were largely or wholly causal then about 25.0% of the deaths in men and 7.6% of deaths in women were due to cigarette/bidi smoking, consistent with findings from Singapore (22.6% for men and 5.3% for women) [25], China (21% for men) [26], and Japan (22% for men and 5% for women). Interestingly, the proportion of all-cause deaths in men attributable to smoking in our study population has reached the level seen in the United States in 2004 [46]. Although there is evidence from prospective studies regarding the benefits of smoking cessation, in terms of decreased risk of mortality from all-cause, cancer and CVD [21], [25], [42], [47]–[52], the magnitude of the gain and the time required to prove results are still debatable. In the present study, a significant reduction in the risk of death from IHD was observed for smokers who have quitted smoking for more than 5 years, a finding supported by studies from other Asian populations [40], [49], [53], [54]. The rapid decline of IHD mortality following smoking cessation has been documented in the Western studies [42], [51], [52]. Within days or weeks, past smokers may experience the benefits of quitting such as increase in platelet activation and change in fibrinolytic system [42]. However, carcinogenic transformation of individual cells and the metastatic growth of the cancer cells are a long process, and reversal of cancer risk will likely to take longer time before full benefits can be observed [42]. In low-income countries such as Bangladesh, the populations are undergoing an epidemiologic transition in which the burden of heart disease is increasing. In addition, environmental risk factors such as air pollution and arsenic exposure that may have synergistic effect on heart disease mortality [12] are more common. Therefore, the promotion of smoking cessation is critically important in these populations.

The strengths of the present study include a population-based prospective design with a high participation rate (97%) at baseline recruitment, the large sample size, and extensive data on tobacco smoking and potential confounding factors collected with the use of in-person interviews at baseline. Although we restricted to married individuals, Bangladesh has the second-highest rate of early marriage [55], and most of the adults are married in rural area. Therefore, we do not believe that the restriction would limit the generalizability of the study. Several potential limitations, however, should also be noted. First, the prevalence of tobacco smoking was very low among women (<10%) compared with men and precluded us from more detailed analyses in women. Second, we did not have data on smokeless tobacco use, a popular habit throughout the world, especially in South and Southeast Asia. According to a 2009 survey, 26% of adult men and 28% of women currently use smokeless tobacco and the proportion of current tobacco users who used both smokeless and smoking tobacco is 22% among men and 3% among women in Bangladesh [56]. Smokeless tobacco use has been shown to increase mortality from all-cause [27], [28], cancer [36], and IHD [57]. To the extent that the use of smokeless tobacco products is positively related to smoking and mortality, the association between smoking and mortality may be overestimated. We were also unable to examine exposure to environmental tobacco smoke, which has been causally related to a variety of health effects such as lung cancer, ischemic heart disease, respiratory effects and other diseases in adults [58]. Mortality attributable to environmental tobacco smoke has also been assessed in several populations, although the results are not entirely consistent [59]–[63]. To the extent that nonsmokers may be exposed to environmental tobacco smoke [58], the association between smoking and mortality may be underestimated. Third, we did not assess the effect of cigarette and bidi smoking on mortality separately. However, bidi smoking has been shown to associate with similar or higher risks of all-cause and cancer mortality compared with cigarette smoking [27], [28], [36] and a bidi delivers a comparable or higher amount of tar and nicotine although a bidi contains a much smaller amount of tobacco (∼0.2 g) than a cigarette (∼1 g) [64].

## Conclusion

Tobacco smoking was associated with increased risks of premature death from all-cause, cancer, and IHD among both men and women in this Bangladeshi population. Substantial proportion of death, especially for men, was attributable to tobacco smoking. Stringent tobacco-control measures and smoking-cessation strategies are needed in Bangladesh as well as other developing countries with a similar setting.

## Supporting Information

Table S1 **Underlying causes of death.**
(DOCX)
