# Supplementary material for: A Prospective Study of Tobacco Smoking and Mortality in Bangladesh
Source: PLoS One. 2013 Mar 11;8(3):e58516. doi: 10.1371/journal.pone.0058516 (PMC3594295; doi:10.1371/journal.pone.0058516)
Supplement: Table S1 — Underlying causes of death. (DOCX) [file pone.0058516.s001.docx]

| **Table S1.** Underlying causes of death. | |
| --- | --- |
| Causes of death (ICD-10 code(s)) | No. of death |
| All causes | 734 |
| Infectious and parasitic diseases (A00-B99) | 49 |
| Tuberculosis (A15-A19) | 27 |
| Other (A09, A35, A41, A82, A91, B18, B19, B90) | 22 |
| Cancer (C00-C97) | 128 |
| Respiratory organs (C30-C39) | 38 |
| Lung (C34) | 31 |
| Larynx (C32) | 7 |
| Digestive organs (C15-C26) | 54 |
| Stomach (C16) | 14 |
| Liver (C22) | 28 |
| Other (C18-C21, C23) | 12 |
| Other | 36 |
| Cardiovascular diseases (I00-I99) | 308 |
| Ischemic heart disease (I20-I25) | 119 |
| Other forms of heart disease (I30-I52) | 43 |
| Stroke (I60-69) | 129 |
| Other (I05, I08, I11, I27) | 17 |
| Respiratory diseases (J00-J99) | 80 |
| Other chronic obstructive pulmonary disease (J44) | 53 |
| Asthma (J45) | 15 |
| Other (J41, J46, J69, J95) | 12 |
| Digestive diseases (K00-K93) | 42 |
| Liver (K70, K72, K74, K76) | 32 |
| Other (K25, K27, K31, K56, K63, K65, K80) | 10 |
| Genitourinary diseases (N00-N99) | 19 |
| Renal failure (N17, N18) | 15 |
| Other (N13, N83, N93) | 4 |
| Symptoms, signs and abnormal clinical and laboratory findings, not elsewhere classified (R00-R99) | 43 |
| External causes of morbidity and mortality (V01-Y98) | 27 |
| Pregnancy, childbirth and the puerperium (O00-O99) | 13 |
| All other causes | 25 |
